# Economic Evaluation of Ultrasound-guided Central Venous Catheter Confirmation vs Chest Radiography in Critically Ill Patients: A Labor Cost Model

**DOI:** 10.5811/westjem.2022.7.56501

**Published:** 2022-09-15

**Authors:** Enyo A. Ablordeppey, Adam M. Koenig, Abigail R. Barker, Emily E. Hernandez, Suzanne M. Simkovich, James G. Krings, Derek S. Brown, Richard T. Griffey

**Affiliations:** * Washington University School of Medicine, Department of Anesthesiology, St. Louis, Missouri; † Washington University School of Medicine, Department of Emergency Medicine, St. Louis, Missouri; ‡ Washington University School of Medicine, St. Louis, Missouri; § Washington University, Center for Health Economics and Policy at the Institute for Public Health, St. Louis, Missouri; ¶ Medstar Health Research Institute, Division of Healthcare Delivery Research, Hyattsville, Maryland; || Georgetown University School of Medicine, Department of Medicine, Washington, DC; # Washington University School of Medicine, Division of Pulmonary Critical Care Medicine, Department of Medicine, St. Louis, Missouri; ** Washington University in St. Louis, Brown School, St. Louis, Missouri

## Abstract

**Introduction:**

Despite evidence suggesting that point-of-care ultrasound (POCUS) is faster and non-inferior for confirming position and excluding pneumothorax after central venous catheter (CVC) placement compared to traditional radiography, millions of chest radiographs (CXR) are performed annually for this purpose. Whether the use of POCUS results in cost savings compared to CXR is less clear but could represent a relative advantage in implementation efforts. Our objective in this study was to evaluate the labor cost difference for POCUS-guided vs CXR-guided CVC position confirmation practices.

**Methods:**

We developed a model to evaluate the per patient difference in labor cost between POCUS-guided vs CXR-guided CVC confirmation at our local urban, tertiary academic institution. We used internal cost data from our institution to populate the variables in our model.

**Results:**

The estimated labor cost per patient was $18.48 using CXR compared to $14.66 for POCUS, resulting in a net direct cost savings of $3.82 (21%) per patient using POCUS for CVC confirmation.

**Conclusion:**

In this study comparing the labor costs of two approaches for CVC confirmation, the more efficient alternative (POCUS-guided) is not more expensive than traditional CXR. Performing an economic analysis framed in terms of labor costs and work efficiency may influence stakeholders and facilitate earlier adoption of POCUS for CVC confirmation.

## INTRODUCTION

Five million central venous catheters (CVC) are inserted in the United States annually.^1^ Following placement of CVCs, confirmation of its position and exclusion of an iatrogenic pneumothorax are typically required for safety prior to use of the catheter for fluid or medication administration. The majority of such confirmation checks are performed by chest radiograph (CXR) at an estimated annual cost of ≥$500 million.^2^,^3^ Emerging literature supports deimplementing the current practice of obtaining a CXR after CVC insertion if point-of-care ultrasound (POCUS) is used to confirm catheter position and exclude a pneumothorax.^4^–^9^ Current standard of care recommends POCUS guidance during CVC insertion.^10^–^12^ Evidence now also supports the use of POCUS for CVC confirmation.^4^–^9^ POCUS-guided confirmation can be rapidly conducted immediately following the POCUS-guided insertion, making practical sense for workflow.

Waiting for CXR to be obtained in a critically ill patient can delay catheter use for delivery of critical medical interventions (ie, antibiotics, vasopressors, etc) and can increase morbidity and mortality.^13^–^19^ Indeed, faster initiation of patient care interventions is the most clear and substantial benefit of POCUS-guided CVC confirmation. The CVC confirmation by CXR traditionally requires 1) a technician to capture the image on a portable CXR machine and 2) a radiologist to interpret the image and bill for the interpretation. In contrast, POCUS-guided confirmation does not require additional equipment or personnel beyond what is required for the insertion itself, does not expose patients to radiation, and can be completed rapidly.^20^–^22^ In addition, use of a POCUS-guided confirmation protocol obviates exposure of additional personnel (the radiology technician) to patients in the context of a pandemic.^23^

Three recent meta-analyses found that POCUS-guided CVC confirmation is feasible, fast, and accurate with diagnostic similarity to CXR confirmation.^13^,^24^,^25^ Yet, POCUS for CVC confirmation has not enjoyed wide adoption for reasons including organizational culture, care delivery routines, and clinical inertia.^26^,^27^ Demonstration of potential cost savings using the POCUS approach would provide additional impetus for its adoption. While cost savings measured by a reduction in CXR have been reported, there has not been an analysis of the costs associated with these CVC confirmation strategies from a personnel and time perspective. We hypothesized that a POCUS-guided CVC confirmation protocol, instead of a CXR protocol, decreases labor costs associated with CVC confirmation.

## METHODS

The cost assessment analysis compared labor costs associated with the standard process (CXR) to the proposed alternative (POCUS) and followed the Consolidated Health Economics Evaluation Reporting Standards (CHEERS) reporting guideline (Supplemental File 1).^28^ The multistep processes of both CVC confirmation techniques are described below.

### Setting

We conducted the study at a large (~1200 hospital beds) academic, urban, residency-affiliated, tertiary care medical center. Chest radiographs are routinely obtained for patients in the emergency department and intensive care units after CVC insertion.

**Population Health Research Capsule**

What do we already know about this issue?

*Millions of chest radiographs (CXR) are performed annually to confirm position and exclude pneumothorax after central venous catheter (CVC) placement.*

What was the research question?

*We evaluated the difference in labor cost of point-of-care ultrasound (POCUS)-guided vs CXR-guided CVC position confirmation practices.*

What was the major finding of the study?

*POCUS-guided confirmation of central line placement is less expensive than chest radiograph ($14.66 vs. $18.48 on average, − 21%).*

How does this improve population health?

*This lower labor cost may facilitate earlier adoption of POCUS for CVC confirmation.*

### Protocol A: Traditional X-ray Confirmation

1. Clinician requests a CXR after CVC placement.
2. Request is received by the radiology department and a technician is sent to the patient’s bedside.
3. The technician performs a digital portable CXR.
4. The radiograph image is then available for the bedside clinician to review. In the absence of an obvious malposition or pneumothorax, the clinician will initiate use of the CVC.
5. The radiograph is interpreted by a radiologist. If evidence of a complication is detected at any point, catheter use may be suspended, and corrective action may be taken.

### Protocol B: The Three-Step Protocol for POCUS-Guided Confirmation

**Figure 1:**
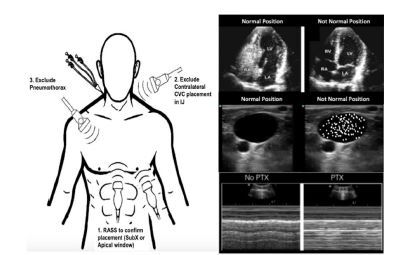
Point-of-care ultrasound-guided catheter confirmation protocol. Image modified from Montrief 2021^3^ *CVC*, central venous catheter; *IJ*, internal jugular; *RASS*, right atrial swirl sign; *SubX*, subcostal view; *LV*, left ventricle; *RV*, right ventricle; *RA*, right atrium; *LA*, left atrium; *PTX*, pneumothorax.

The POCUS-guided protocol evaluates the CVC position using three steps performed by the clinician placing the CVC (Figure 1): confirm venous placement; rule out catheter malposition; and rule out pneumothorax.^13^,^14^,^24^,^29^–^31^

1. Obtain a subcostal or apical four-chamber view of the heart while an assistant rapidly injects 10 milliliters of normal saline into the distal catheter lumen, confirming placement in or near the superior vena cava if turbulent flow, known as the “swirl sign”, is observed in the right atrium within two seconds of catheter flush.^32^
2. Obtain a view of the patient’s neck vessels (internal jugular and carotid) contralateral to the catheter location, and the assistant rapidly injects saline. A swirl sign should not be observed in the internal jugular or carotid during this step. If present, this may indicate catheter tip malposition.^13^,^24^,^25^
3. Obtain a mid-clavicular view of the pleura on the same side of the chest relative to the catheter location to demonstrate lung slide and exclude a pneumothorax.^33^ Visualization of pleural movement medial and lateral to the mid-clavicular point excludes an anterior pneumothorax.^34^,^35^

### Model Description

**Figure 2:**
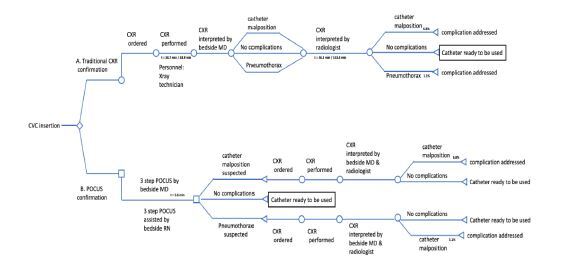
A decision tree model comparing protocol A (traditional X-ray confirmation) vs protocol B (POCUS-guided confirmation). *CVC*, central venous catheter; *CXR*, chest radiograph; *POCUS*, point-of-care ultrasound; *MD*, medical doctor.

We constructed a decision tree-based model (Figure 2) from current practice for CVC confirmation, comparing CXR-guided to the proposed three-step POCUS-guided confirmation protocol. Modeling assumptions are made explicit in the text below and were tested using sensitivity analyses. See Table 1 for model variables. We used personnel costs in each protocol based on the common practice at our institution, and their roles were defined by standard processes at our local institution. Median salary data (total cash compensation) for relevant specialties (emergency medicine, critical care medicine, surgery, radiology) and ranks were obtained from the Association of American Medical Colleges list of large, research-intensive academic medical centers.^36^ Faculty physician salaries were assumed to compensate for approximately a 60-hour work week.^37^–^39^ To focus the model on billable labor costs associated with POCUS we did not use the salaries of physicians in training and advance practice practitioner in the model. For registered nurses and radiology technicians, wage rates were taken from the Bureau of Labor Statistics for those occupations working in hospital settings.^40^,^41^ We then integrated labor costs per unit time with time data to quantify actual labor costs for each segment of the decision tree.

Table 2a demonstrates probability variables based on both internal and external data.^24^,^25^,^42^ We conducted one-way and two-way sensitivity analyses based on input from the literature about process steps within the protocols (Table 2b).^24^ Salary ranges are based on 25^th^ and 75^th^ percentiles from national sources while the times are based on reported standard deviation when available and estimated based on experience of practicing clinicians. Sensitivity analyses were not performed for salary data as these figures should be distributed equally across protocols and should change proportionally in other settings. In addition, we account for the potential for some cases in the POCUS-guided protocol to be diverted back to routine care (traditional CXR) after an unsuccessful attempt to confirm catheter position by POCUS. We make the assumption that ultrasound and CXR machines at our institution will be retired due to obsolescence before they are retired due to wear and tear and that changes in usage will not alter maintenance schedules. Thus, we did not examine costs associated with equipment purchase or maintenance. Furthermore, we did not measure the cost of training operators (or radiograph technicians) or disposable equipment.

## RESULTS

The labor cost per patient from our model using protocol A (CXR) was $18.48, while the expected labor cost per patient under protocol B (POCUS) was $14.66. The estimated cost savings, in labor, for switching to protocol B is $3.82 (21%) per patient (Table 3). The primary driver of savings was replacing the radiology technician labor costs with nursing labor costs in the POCUS-based protocol. Cumulative physician labor costs were also less in the POCUS-based protocol due to slightly less overall time required (radiologist plus bedside physician) and payment differential for bedside physicians vs radiologists. A portion of the cost savings with the CXR-based protocol was negated by the possibility of patients in the POCUS-based protocol diverting to a CXR due to suspected complications seen on POCUS. We estimate that 7.9% of patients are diverted to CXR during the three-step protocol.^25^ Still, the costs saved on care of the remaining 92% of POCUS-protocol patients outweigh the additional cost of diverted patients.

In our institution, there were 3,069 CVC placements in one year, approximately 2,045 of which used a CXR for catheter position confirmation and pneumothorax exclusion.^43^ Thus, the cost of protocol A using CXR to confirm CVC was $37,792 compared to the cost of $29,984 if we used protocol B with POCUS guidance. For our hospital, this would result in a savings of $7,808 per year. Of the five million CVCs placed annually in the US, we estimate that 66%, or 3.3 million, are supradiaphragmatic CVCs eligible for POCUS-guided confirmation.^43^ Generalizing these costs across the entire US healthcare system with 3.3 million eligible CVCs per year, the cumulative labor costs of protocol A (CXR-based) are $61.0M vs $48.4M for protocol B (POCUS-based). By making the transition to using POCUS, there would be estimated savings (from labor cost only) to the US healthcare system of $12.6 million annually.

### Sensitivity Analysis

**Figure 3:**
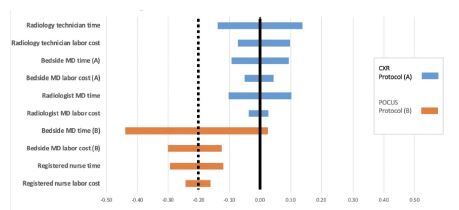
Tornado chart depicting percent change in total cost with variation in individual variables. Each bar depicts deviation in the total cost of a protocol that follows variation of an individual variable. *MD*, medical doctor; *CXR*, chest radiograph; *POCUS*, point-of-care ultrasound.

We assessed two-way sensitivity using a tornado diagram (Figure 3). Our sensitivity analysis revealed a robust cost savings that persists at the extremes of most variables (Supplemental Tables 1 and 2). The exception is that protocol B (POCUS based) would be 3% more costly at the upper extreme of bedside physician time. Ultimately, our model strongly suggests that implementation of protocol B would result in lower labor costs in the vast majority of scenarios.

## DISCUSSION

Rising healthcare costs in the US necessitate that health systems identify opportunities to optimize resources such as labor-associated costs during patient care.^44^ Our findings suggest that POCUS is faster and has associated workflow-efficiency benefits, and that regarding labor costs the use of CXR for CVC confirmation is slightly more expensive compared to POCUS. Other studies have looked at equipment cost, 22,45,46 but to our knowledge our study is the first cost-comparison study to evaluate the organizational labor costs of POCUS-guided CVC confirmation compared to standard of care (CXR). Labor costs are more informative for such decisions, as radiographs and ultrasound are readily available in large academic medical centers and thus are not marginal costs to consider. While on an individual basis, the cost differences are marginal, these values become more substantial when considering the annual average number of CVC insertions performed. Our data also suggests that POCUS-guided CVC confirmation decreases time to initiate care, which can yield improvements in patient safety and further improve internal efficiencies and lower costs.

In our sensitivity analyses, varying time assumptions consistently yielded cost savings using the POCUS strategy, indicating that the results of our modeling appear to be robust and that savings occur at extremes of time and salary for the majority of variables. Our analysis used faculty salary data and did not account for the possibility of trainees (residents) or advanced practice practitioners conducting the POCUS-based protocol, which would further lower costs. Chui et al reported that healthcare costs associated with CXRs after CVCs are high and had an excessive number needed to treat suggesting that postprocedural CXR is an expensive screening test. In their study, 286 CXRs would be needed after POCUS-guided right internal jugular vein CVC to detect one additional malpositioned catheter requiring intervention and 866 CXRs would be needed to detect a pneumothorax that required tube thoracostomy.^7^ We have a similar incidence of catheter malposition and pneumothorax at our institution suggesting similar numbers of CXRs needed to prevent one CVC-related mechanical complication requiring intervention.^43^,^47^ Alternatively, Hirshberg et al used billing data to estimate a hypothetical hospital-wide cost savings of $54,494 per year by using POCUS for CVC confirmation instead of CXR, suggesting that whether measuring by facility cost, billing data, or labor cost, using POCUS is associated with a cost savings.^48^ Our data suggests that in addition to these other cost perspectives, from a labor cost point of view, POCUS is less costly than CXR for CVC confirmation.

Secondary safety improvements achieved using POCUS-guided CVC confirmation are harder to quantify but are likely to reduce costs. Most notably, facilitating earlier patient care initiatives (using the CVC for its intended purpose) results in better outcomes for high-acuity patients. For example, it is estimated that delayed vasopressor administration in cardiac arrest or sepsis translates to a 10% per minute decline in the odds of hospital discharge with a favorable outcome.^49^–^51^ Using POCUS as the first-line screening for CVC-related mechanical complications accepts a higher rate of false positives for patient safety. In this way the benefit of earlier medical management after CVC confirmation is present, and delays associated with CXR are avoided in most patients. When mechanical complications are a possibility (minority of patients), the delay is accepted and a CXR is necessary to determine whether intervention is needed. As reported in the literature, most mechanical complications (malposition, pneumothorax) in fact do not require reposition or tube thoracostomy. Other safety benefits of POCUS include limiting exposures for patients and technicians using POCUS rather than CXR confirmation and reducing the risk of transmission of infectious agents (including COVID-19) and the propagation of nosocomial infections.^52^,^53^ And notably, CXR exposes patients to ionizing radiation (albeit low level) while POCUS does not.

Finally, POCUS-guided CVC confirmation seen in our study can streamline physician workflow and significantly improve internal efficiency. The POCUS protocol’s linear workflow avoids the need to switch between unrelated tasks. A clinician can place a CVC, confirm placement, and initiate care all in one sitting without leaving the patient’s bedside. In contrast, the CXR confirmation protocol leaves significant time between completion of CVC placement and completion of the CXR, thus requiring the clinician to task switch during downtime before returning to the task of confirming CVC placement and initiating care.^24^ Task-switching is known to increase error rates,^54^,^55^ and is estimated to contribute to costs of over $280 million per year in the US.^56^ Ultimately, physician workflow during CVC confirmation can also be improved by eliminating CXR when POCUS has already been used to confirm the CVC. One survey of emergency and critical care physicians found that many already use POCUS to evaluate for pneumothorax (15% always; 58% sometimes) or catheter misplacement (20% always; 49% sometimes).^27^ Reducing this redundancy during CVC confirmation and using POCUS alone as a first-line screen will likely reduce the number of CXRs needed and associated costs.

## LIMITATIONS

Our study has several limitations. We conducted this study at a single-center, large, urban, academic trauma center. Cost differences observed will likely vary by setting. Our analysis is based on labor costs only, not accounting for professional or facility charges associated with either protocol. In addition, our analysis relies on modeling, which by definition implies the simplification of reality, and simplifying assumptions were made in the model presented.^57^ Our model makes the assumption that training costs of the following variables would not change from standard operating costs in either protocol: 1) training for a clinician to use POCUS to insert CVCs and interpret CXR; and 2) training a clinician to use POCUS to insert and confirm a CVC.

Our analyses are calculated and projected, as standard care at our hospital currently uses CXR for CVC confirmation. Values are based on probability and not actual costs at our institution. We did make efforts to minimize bias by providing comprehensive assessments and analysis that would most mimic our local environment. We did not measure opportunity costs (nor implementation cost) associated with a new POCUS-guided CVC confirmation protocol. For example, our analysis assumes that a POCUS machine is widely available, training in bedside diagnostics is present, and a high number of CVC insertions occur annually. Finally, this was not a cost effectiveness analysis. We believe labor costs alone comprise a sufficient portion of the overall cost to allow inferences that the overall costs per patient would be lower. However we cannot make this case with absolute certainty. Further investigation would involve a more robustly defined measure of effectiveness. Although we evaluated the healthcare cost to the health system, there are other benefits of POCUS-guided CVC confirmation not captured in our analysis: less radiation exposure and quicker utilization of the catheter, for example, which have potentially greater value than just cost savings to the healthcare system.

## CONCLUSION

Our study found modest labor cost savings by using point-of-care ultrasound to confirm central venous catheter position and exclude pneumothorax in the emergency department and intensive care unit. In addition to features of the POCUS approach such as time savings and workflow efficiency, which also likely have cost implications, labor cost is another consideration conferring an advantage to this approach to CVC confirmation and may serve as a facilitator to its adoption. Future studies should characterize the resource implications of substituting POCUS-guided CVC confirmation more fully by conducting a comprehensive assessment of the costs of development, implementation, and maintenance of this change in process.

## Supplementary Information





## Figures and Tables

**Table 1:** Model variables.

Parameter	Central Estimate	Range Used in Sensitivity Analysis
Bedside MD time per Protocol A	3.0 minutes	2 – 4
Bedside MD time per Protocol B	5.6 minutes	3.1 – 8.1
Radiology MD time for radiograph interpretation	3.0 minutes	2 – 4
RN time per Protocol B	5.6 minutes	3.1 – 8.1
Radiology technician time per Protocol A	15.0 minutes	10 – 20

Bedside MD labor cost ($/minutes)*	1.72	1.41 – 1.99
Radiology MD labor cost ($/minutes)*	1.89	1.66 – 2.06
RN labor cost ($/minutes)**	0.64	0.52 – 0.79
Radiology technician labor cost ($/minutes)**	0.51	0.42 – 0.63

*MD*, medical doctor; *RN*, registered nurse.

* MD labor costs per minute were determined using annual salary estimates and dividing by estimated annual minutes.

Our sensitivity analysis used annual salary as a fixed variable and calculated pay ranges using a range of annual minutes worked.

** RN and radiology technician labor costs and ranges were taken directly as hourly pay and converted to pay in minutes.

**Table 2:** Complication probability (2a) and time (2b) variables of chest radiograph and point-of-care ultrasound use for catheter confirmation

a. Complication Incidence				

Detection Method	Complication	Meta-Analysis, Smit 2018 25	Meta-Analysis, Ablordeppey 2017 24	Internal (Ablordeppey, 2019)
Radiology Interpretation of CXR	(+) Malposition	6.80%	17.60%	2.60%
Radiology Interpretation of CXR	(+) PTX	1.10%	1.10%	3.20%

b. Time Intervals				

Interval Start	Interval End	Meta-Analysis, Smit 2018 25	Meta-Analysis, Ablordeppey 2017 24	Internal (Ablordeppey, 2019)

CXR ordered	CXR performed	34.7 min [32.6–36.7]	63.9 min ± 57.1	29 min [1–269]
CXR ordered	Radiology read complete	46.3 min [44.4 – 48.2]	143.4 min ± 123.7	
POCUS confirmation initiated	POCUS confirmation complete	2.83 min [2.77 – 2.89]	5.6 min ± 2.5	9 min [8.5 – 9.5]

*CXR*, chest radiograph; *POCUS*, point-of care ultrasound; *PTX*, pneumothorax; *min*, minute.

**Table 3:** Cost comparison between Protocol A versus B.

Variable	Protocol A (CXR) Costs	Protocol B (POCUS) Costs	Cost Difference
Cost of uncomplicated confirmation	CXR by radiology technician 15 minutes × $0.51/minute = $7.65 CXR review by bedside MD 3 minutes × $1.72/minute = $5.16 CXR review by radiologist 3 minutes × $1.89/minute = $5.67	POCUS confirmation by bedside MD 5.6 minutes × $1.72/minute = $9.65 POCUS confirmation assisted by bedside RN 5.6 minutes × $0.64/minute = $3.57	− $5.26
Cost of diverting to CXR protocol due to malposition	-	0.068^1^ × $18.48 = $1.26	
Cost of diverting to CXR protocol due to pneumothorax	-	(1−0.068) × 0.011 × $18.48 = $0.19	+ $1.45
Total cost per patient	$18.48	$14.66	− $3.82 (−21%)
Estimated annual total cost for hospital (n = 2045)^2^	$37,792	$29,984	− $7,808
Estimated cost per 1 million CVCs	$18.5M	$14.7M	− $3.8M

*CVC*, central venous catheter; *CXR*, chest radiograph; *POCUS*, point-of-care ultrasound; *MD*, medical doctor; *RN*, registered nurse.

1 From Smit meta-analysis, 2018;

2 From Ablordeppey internal data, 2019.
