# Supplementary material for: Economic Evaluation of Ultrasound-guided Central Venous Catheter Confirmation vs Chest Radiography in Critically Ill Patients: A Labor Cost Model
Source: West J Emerg Med. 2022 Sep 15;23(5):760–8. doi: 10.5811/westjem.2022.7.56501 (PMC9541994; doi:10.5811/westjem.2022.7.56501)
Supplement: Supplementary file 1 [file wjem-23-760-s001.docx]

Supplemental File 1. Sensitivity analysis of cost comparison between Protocol A versus B, using high and low time estimates

| **Variable** | **Protocol A (CXR)** | | **Protocol B (POCUS)** | |
| --- | --- | --- | --- | --- |
|  | **Low Time Estimate** | **High Time Estimate** | **Low Time Estimate** | **High Time Estimate** |
| Costs of uncomplicated confirmation | CXR performed by radiology technician  10 minutes × $0.51/minute **= $5.10**  CXR review by bedside MD  2 minutes × $1.72/minute **= $3.44**  Review by radiologist  2 minutes × $1.89/minute **= $3.78** | CXR performed by radiology technician  20 minutes × $0.51/minute **= $10.20**  CXR review by bedside MD  4 minutes × $1.72/minute **= $6.88**  Review by radiologist  4 minutes × $1.89/minute **= $7.56** | POCUS confirmation by bedside MD  3.1 minutes × $1.72/minute = **$5.33**  POCUS confirmation assisted by bedside RN  3.1 minutes × $0.64/minute **= $1.98** | POCUS confirmation by bedside MD  8.1 minutes × $1.72/minute **= $13.93**  POCUS confirmation assisted by bedside RN  8.1 minutes × $0.64/minute **= $5.18** |
| Cost of diverting to CXR protocol due to malposition | **-** | **-** | 0.068*^1^* × $12.32 **=**  **$0.84** | 0.068*^1^* × $26.64 **=**  **$1.68** |
| Cost of diverting to CXR protocol due to pneumothorax | **-** | **-** | (1-0.068) × 0.011 × $12.32 **= $0.13** | (1-0.068) × 0.011 × $24.64 **= $0.25** |
| Total cost per patient | **$12.32** | **$24.64** | **$8.28** | **$21.04** |
| Estimated annual total cost for hospital (n=2045)*^2^* | **$25,194** | **$50,389** | **$16,933** | **$43,035** |
| Estimated cost per 1 million CVCs | **$12.3M** | **$24.6M** | **$8.3M** | **$21.0M** |

CVC = central venous catheter; CXR = chest x-ray; POCUS = point of care ultrasound; PTX = pneumothorax; MD = medical doctor; RN = registered nurse

^1^ From Smit meta-analysis, 2018

^2^ From Ablordeppey internal data, 2019
