# Supplementary material for: Economic Evaluation of Ultrasound-guided Central Venous Catheter Confirmation vs Chest Radiography in Critically Ill Patients: A Labor Cost Model
Source: West J Emerg Med. 2022 Sep 15;23(5):760–8. doi: 10.5811/westjem.2022.7.56501 (PMC9541994; doi:10.5811/westjem.2022.7.56501)
Supplement: Supplementary file 2 [file wjem-23-760-s002.docx]

Supplemental File 2. Sensitivity analysis of cost comparison between Protocol A versus B, using high and low salary estimates

| **Variable** | **Protocol A (CXR)** | | **Protocol B (POCUS)** | |
| --- | --- | --- | --- | --- |
|  | **Low Salary Estimate** | **High Salary Estimate** | **Low Salary Estimate** | **High salary Estimate** |
| Cost of uncomplicated confirmation | CXR performed by radiology technician  15 minutes × $0.42/minute **= $6.30**  CXR review by bedside MD  3 minutes × $1.41/minute = **$4.23**  Review by radiologist  3 minutes × $1.66/minute = **$4.98** | CXR performed by radiology technician  15 minutes × $0.63/minute **= $9.45**  CXR review by bedside MD  3 minutes × $1.99/minute **= $5.97**  Review by radiologist  3 minutes × $2.06/minute **= $6.18** | POCUS confirmation by bedside MD  5.6 minutes × $1.41/minute = **$7.90**  POCUS confirmation assisted by bedside RN  5.6 minutes × $0.52/minute **= $2.91** | POCUS confirmation by bedside MD  5.6 minutes × $1.99/minute **= $11.14**  POCUS confirmation assisted by bedside RN  5.6 minutes × $0.79/minute **= $4.42** |
| Cost of diverting to CXR protocol due to malposition | **-** | **-** | 0.068*^1^* × $15.51 **=**  **$1.05** | 0.068*^#^* × $21.60 **=**  **$1.47** |
| Cost of diverting to CXR protocol due to pneumothorax | **-** | **-** | (1-0.068) × 0.011 × $15.51 **= $0.16** | (1-0.068) × 0.011 × $21.60 **= $0.22** |
| Total cost per patient | **$15.51** | **$21.60** | **$12.02** | **$17.26** |
| Estimated annual total cost for hospital (n=2045)*^2^* | **$31,718** | **$44,172** | **$24,580** | **$35,297** |
| Estimated cost per 1 million CVCs | **$15.5M** | **$21.6M** | **$12.0M** | **$17.3M** |

CVC = central venous catheter; CXR = chest x-ray; POCUS = point of care ultrasound; PTX = pneumothorax; MD = medical doctor; RN = registered nurse

^1^ From Smit meta-analysis, 2018

^2^ From Ablordeppey internal data, 2019
